# An integrative framework for drug target discovery bridging clinical trial and genetic data insights

**DOI:** 10.1186/s12967-026-07806-x

**Published:** 2026-03-13

**Authors:** Johanna Mielke, Tobias Strunz, Jeongah Lee, Patrick Schloemer, GCKD Investigators, Giuseppe Gallone

**Affiliations:** 1 Translational Sciences, Bayer AG, Aprather Weg 18a, 42113, Wuppertal, Germany; 2 Clinical Statistics & Analytics, Bayer AG, Berlin, Germany

**Keywords:** Chronic kidney disease, Disease progression, Precision medicine, Target identification, Backtranslation

## Abstract

**Background:**

Over the past few years, the considerable growth in the availability of population-scale genomic data has provided a significant boost in supporting quantitative, well powered, data-driven approaches to drug target discovery. However, population-scale genomic biobanks often lack comprehensive longitudinal phenotyping and in-depth clinical annotation. In contrast, clinical trial data, rich in phenotypic detail, frequently lack accompanying omics information, hindering mechanistic understanding of clinical findings. To address these shortcomings, we propose a framework called “back-translation” that leverages the strengths of both datasets by translating patient insights from clinical data to biobank context, to enable the discovery of novel insights based on the unique strengths of both data types.

**Methods:**

Our framework consists of two main steps. First, we identify a subgroup of interest within the clinical data and construct a classifier (risk score) to accurately identify patients in this subgroup. In the second step, we validate the derived risk score and then transfer it to the biobank data. The risk score serves as a proxy for characterizing the subgroup, which enables us to perform rare and common genetic variant association tests.

**Results:**

We demonstrate the value of this approach in a pilot study using clinical trial data from the FIDELITY dataset combined with biobank data from the UK Biobank (UKBB) and the German Chronic Kidney Disease (GCKD) cohort, focusing on fast kidney disease progression in patients with Chronic Kidney Disease (CKD). Our results show that the derived risk score accurately identifies high-risk patients in both FIDELITY and GCKD. Our genetic analysis of the clinical risk score in the UKBB identifies multiple genes that may serve as candidates for novel therapeutic target investigation.

**Conclusion:**

We propose a generalizable framework for the identification of data-driven targets that is therapeutic area-agnostic. This approach offers a novel and innovative opportunity to integrate clinical data into target identification via “back-translation,” utilizing clinical insights previously underutilized in a research context. By bridging clinical and genetic data, our framework enhances the potential for discovering novel therapeutic targets and for advancing precision medicine.

**Trial registration:**

NCT02540993, NCT02545049

**Supplementary Information:**

The online version contains supplementary material available at 10.1186/s12967-026-07806-x.

## Background

Precision medicine holds significant promise for the advancement of therapeutic strategies in a wide range of diseases  [1]. However, there is a striking gap between the identification of clinically defined subgroups (i.e. a group of patients characterized by a specific set of biomarkers) and the development of targeted therapeutic interventions, primarily due to a lack of understanding of some or all of the underlying disease mechanisms. In this context, genetic data emerges as a crucial resource, providing a data-driven approach to uncovering potential therapeutic targets  [2]. In recent years, the release of large-scale genetic data sets, such as UK Biobank (UKBB)  [3], has enabled researchers to identify targets with strong human genetic support based on an unprecedented wealth of genomic information via high-quality large-scale sequencing for a large, relatively unbiased population cohort. However, while genetic data offer rich insights, patient phenotyping is often incomplete or inaccurate  [4]. In the era of precision medicine, a detailed characterization of patients is crucial for identifying subpopulations of interest requiring targeted therapies. On the other hand, clinical trial data presents a complementary set of strengths and weaknesses. While clinical trials, especially in Phase II and III, are rich in phenotypic detail, providing a wealth of information on patient characteristics, treatment responses, and outcomes, they almost always lack accompanying -omics data, such as transcriptomic, proteomic and, crucially, genomic or genetic information. The absence of molecular data hinders the ability of researchers to perform exploratory analyses to derive mechanistic understanding from the clinical findings, such as, for example, what are the molecular drivers of subpopulations of interest, making it difficult to translate any observations into actionable insights for drug development.

To address these challenges, we propose a novel framework called “back-translation” that takes advantage of the complementary strengths of both biobank and clinical trial data to identify drug targets for patient populations of interest. By translating insights gained from clinical data to a biobank context, our framework facilitates an integrative approach to drug target identification and validation. This combined approach not only enhances the understanding of disease mechanisms but also allows for the identification of new therapeutic target hypotheses that may have previously been overlooked due to clinical observations not being translatable onto the molecular level of pathogenesis.

Our framework consists of two core steps. First, we identify a subgroup of interest within the clinical data and construct a classifier to accurately identify patients within this subgroup. This classifier enables us to focus on specific patient populations that exhibit particular characteristics relevant to the disease of interest, for example, subjects with a high medical need (e.g., a fast progression of disease). In the second step, we translate the derived risk score to the biobank data, which allows us to create a proxy for the subgroup of interest. The risk score is then used to match the clinical characteristics of interest, based on the clinical trial data, to the omics layers, which are only available in the biobank component of the framework. We use both variant-based and gene-based genotype-to-phenotype association analyses to identify loci and genes that are statistically associated with the risk score. The results of the genetic analyses are post-processed to generate an initial candidate set of therapeutic targets. The approach is described in detail in Sect. 2.

To illustrate the utility of our approach, we conducted a pilot study using clinical trial data from the FIDELITY dataset (Finerenone, combined data from FIDELIO-DKD and FIGARO-DKD)  [5, 6] in conjunction with biobank data from the UKBB and the German Chronic Kidney Disease (GCKD) Cohort. In the pilot study, we focused on patients with a high risk of rapid kidney disease progression, a high medical need. Our results show that the derived risk score accurately identifies high-risk patients in both the FIDELITY and GCKD data. Finally, we used the UKBB to conduct genetic analyses and identified multiple genetic loci of interest. The results are described in Sect. 3.

## Framework and methods

### Translating clinical trial results to biobank data: a generalizable approach

Here, we provide a high-level overview of the approach we propose, and we describe it in more detail via a concrete example: an analysis of fast progressors sub-population in Chronic Kidney Disease (CKD) patients. Detailed information about all methods and parameters we applied can be found in the Supplement.

As a starting point, we assume that there exists a patient subpopulation of interest. We define a subpopulation as group of individuals which is identified by a specific biomarker profile and shows a distinct disease trajectory. The aim of the analysis is to understand the genetic underpinnings of the existing, but not yet mechanistically well-characterized, subgroups.

**Fig. 1:**
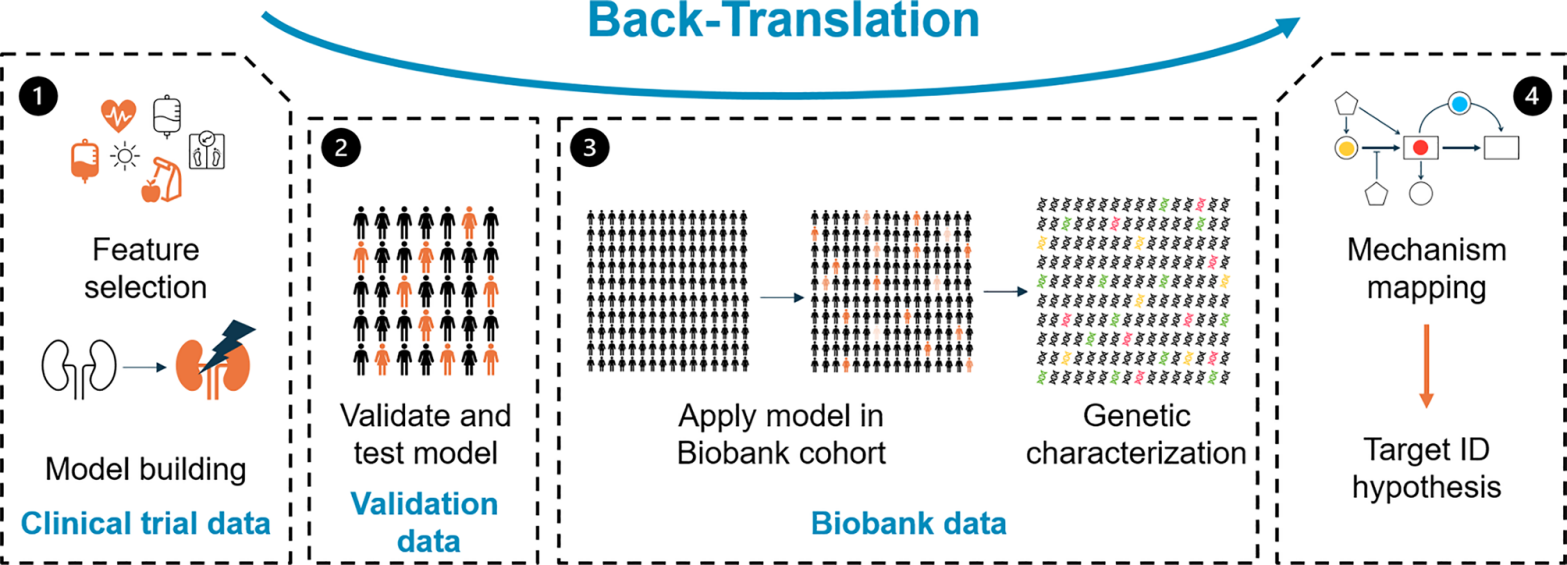
Flowchart of the back-translation framework. The approach starts by building a model in clinical trial data (*panel 1, left*), proceeds to validation (*panel 2, center-left*) and then further to the actual translation to biobank data (*panel 3, center-right*). The final step (*panel 4, right*) focuses on result interpretation

We conceptually divide the framework we propose into four steps, which we outline in Fig. 1.

In Step 1, we construct a model that predicts subjects who are part of the subpopulation of interest based purely on clinical variables. In order for our approach to be viable, all variables that are part of the candidate set of clinical variables need to be available in the biobank data, too. Further, we limit model-building to variables recorded at baseline in the study, as longitudinal data is sparse in biobanks. These constraints are the reason why we found that simply using a published risk score for the task was not an option. The specific choice of model depends on the individual use case: if we model a subpopulation of interest, which may be derived based on a clustering approach, a logistic regression model or a decision tree may be appropriate. On the other hand, if we, for example, focus on fast progressors of disease, we can use a Cox model  [7] to incorporate the time dimension into the model.

Step 2 focuses on the characterization and validation of the risk score. The validity of the framework is closely related to the ability of the derived risk score to identify the subpopulations of interest not just in the clinical trial data, but also in the biobank data. That is why it is important to assess the performance of the model in both the clinical trial data as well as in an external validation dataset. The validation data set can be the same as the biobank data that is used for the genetic assessment, but will typically be an additional dataset since the phenotype information in the biobank may be too sparse (which motivates our approach). Only if the validation leads to satisfactory results it is reasonable to move to Step 3. It should be noted that external validation is highly desirable, but technically not always feasible: for example, if non-responders to a treatment in development or shortly after market access are studied, finding biobank data with subjects on the experimental drug might be tricky or impossible, as it takes time for a drug to be used in clinical practice, even after approval. In these cases, one may skip the external validation. An internal validation within the clinical dataset may be opportune.

In Step 3, the prediction model is evaluated in the biobank data. A risk score is calculated for each subject in the biobank data. Then, the risk score is correlated with the genetic profile of the biobank participants. We propose to screen for both ultra-rare coding as well as genome-wide common germline variation by conducting genome-wide association studies (GWAS) and gene-collapsing burden tests of rare, likely pathogenic or predicted deleterious coding variants, if available.

Step 4 aims to identify potential causal molecular mechanisms for the phenotype of interest. For this purpose, all genomic regions are assigned to genes, which are then classified into pathways and mechanisms. Any resulting candidates can be used as a starting point for the identification of novel therapeutic targets associated with the investigated phenotype.

### Case study: fast progressors in CKD

In our case study we focus on fast progressors in CKD. CKD is a chronic condition that is characterized by a gradual loss of kidney function. It is a major public health concern around the world  [8]. Due to the increasing prevalence of diabetes and hypertension and unhealthy lifestyles, the prevalence of CKD is expected to increase, with more than 800 million people affected in 2022  [9]. Although multiple treatment options for CKD are available (some, such as Finerenone  [10], were only introduced recently), there is still a high residual risk of kidney disease progression and cardiovascular comorbidity  [11].

#### Clinical study and biobank data

We identified a subpopulation (i.e, subjects with fast progression of kidney disease) and constructed the model based on clinical trial data from the FIDELITY dataset  [12] which is a combined dataset of two randomized, double-blind, placebo-controlled, multi-centre clinical trials for Finerenone: FIDELIO-DKD and FIGARO-DKD  [5, 6]. In this prespecified, combined dataset with 12,990 participants, eligible patients were adults (aged 18 years and older) with type 2 diabetes and CKD (urine albumin-to-creatinine ratio $\ge 30- < 300$mg/g and eGFR $\ge 25- \le 90$ ml/min/1.73*m*^2^ or urine albumin-to-creatinine ratio $\ge 300- < 5,000$mg/g and eGFR $\ge 25$ ml/min/1.73*m*^2^) who received the highest tolerated labeled dose of an angiotensin-converting enzyme inhibitor or an angiotensin receptor blocker. We focus on the arm without experimental treatment (that is, the placebo arm, $N=6,492$ patients). One subject without any biomarker data at baseline was excluded, leaving an analysis set of $N=6,491$ patients. Extensive biomarker measurements and survival outcome data were collected during the 3-year study follow-up period. The studies were approved by international review boards and, following the national and international regulations, independent ethics committees and competent authorities. Informed consent was provided by all participants.

We validated the model in the German Chronic Kidney Disease (GCKD) study. The study has been described in detail elsewhere  [13]. In short, this is a national cohort study with 5,217 individuals (1,868 of which having diabetes at baseline) who were recruited between 2010 and 2012. Participants were genotyped, deeply phenotyped at baseline, and followed for six years. During the follow-up period, information on disease progression (e.g. hospitalization for renal and cardiovascular diseases) was collected. Details on inclusion criteria and phenotyping of participants are reported in the Supplement. Due to its relatively small ($N=5,217$) sample size and ensuing power considerations, we did not consider using the GCKD cohort directly for genetic assessment, preferring the UKBB instead.

UKBB is a prospective cohort study that recruited approximately 500,000 volunteers aged between 40 and 69 in the UK. Details on the study can be found elsewhere  [3]. In summary, patients were recruited from 2006 to 2010 and extensively genotyped and phenotyped at the beginning of the study. More details on phenotypes and genotyping, including pre-processing steps, can be found in the Supplement. Longitudinal UKBB phenotyping is relatively sparse: it is based primarily on hospital records (ICD-coded), and it is known to be depleted of CKD diagnoses  [14]. In addition, most UKBB molecular biomarkers are available only on the date of first recruitment. Based on this, we reasoned that using CDK diagnoses in the UKBB directly would have hindered our ability to maximise the power of the genotypic data in pointing us towards novel target hypotheses, which led us to pursue this clinical model to biobank data mapping approach.

Before running any analyses, we aligned units of measurement across datasets and scaled the data to allow for translation of models between datasets. Further details on data alignment considerations can be found in the Supplement.

#### Tailoring the workflow to CKD fast progressors

##### Step 1: construction of a classifier to identify the subpopulation of interest

In our use case, we defined the subgroup of interest as subjects who reach the primary endpoint of the FIDELIO-DKD study, which is the composite outcome of the time-to-first onset of kidney failure (end-stage kidney disease, i.e., initiation of long-term dialysis (for ≥ 90 days) or kidney transplantation or an eGFR of less than 15 ml per minute per 1.73 *m*^2^), a sustained decrease (i.e., two measurements with at least four weeks difference) of at least 40% in the eGFR from baseline  [15] or death from renal causes  [5]. Our analysis focused on subjects in the placebo arm. For model building and internal validation, we split the data randomly in training (2/3 of subjects) and test (1/3 of subjects).

Let $Y_j, j=1, \dots, k,$ be random variables that represent the measured clinical variable *j*. Then we define for each subject $i, i=1, \dots, N,$ the actual clinical measurements as *y*_*ij*_. As in the primary analysis of the clinical study, we used the time to outcome which is given by $t_i, i=1,\dots,N$ for the subject *i* and construct in addition an indicator variable $I_i, i=1,\dots,N$, which gives for subject *i* the information on whether the event was observed or censored. We only included clinical biomarkers that were also available in the biobank datasets to ensure the translatability of the risk score. A full list of included markers, with availability information in UKBB and GCKD, is provided as a Supplementary Table (Supplementary material 2). As a sensitivity analysis, we also constructed one model with the full set of available biomarkers in FIDELITY with the aim to demonstrate that there is no substantial decrease in performance of the model if we restrict to the intersection of available markers (details can be found in the Supplementary Material). Our aim was to maximize the prediction of outcome (i.e. the subpopulation of fast progressors) given available clinical biomarkers.

Variable selection and model building follow a stepwise approach: first, we calculated univariate Cox regression models  [7], including one of the clinical biomarkers of interest, $Y_j, j=1,\dots,k,$ and in addition age and sex and study ID, i.e. we modeled hazard ratios by

1 $$\lambda_j(t|Y_j)=\lambda_{0,j}(t) \cdot exp(\beta_{1,j} y_{ij}+\beta_2 a_i + \beta_3 s_i+ \beta_3 D_i),$$

where *a*_*i*_ gives the age and *s*_*i*_ the sex and *D*_*i*_ the study ID of subject *i*.

We extracted the P-values *P*_*j*_ of the point estimates for $\beta_{1,j}$ and defined a set *S* that contained all clinical biomarkers *j* with a P-value *p* < 0.1:

2 $$S=\bigcup_{j: where P_j < 0.1} Y_j. $$

We then proceeded with a variable selection step. We aimed to construct a parsimony model with maximal predictive power to allow for a reasonably simple translation to the biobank data. We decided on a forward selection procedure, in which we started with an empty Cox regression model and sequentially added additional clinical biomarkers one by one based on the Bayesian Information Criterion (BIC)  [16]. We stopped if the BIC did not improve after the addition of more variables, called the final set of variables $S^*$, and estimated the final Cox model. Let $\hat{\beta}_j$ be the estimated coefficient in the final Cox variable for the clinical variable *Y*_*j*_. Then, the risk score for the subject *i* was defined as

3 $$R_i= \sum_{j \in S^*} \hat{\beta}_j y_{ij}.$$

We constructed two separate models: one based exclusively on non-urine (i.e., blood, body composition) biomarkers and one based on the full set of overlapping biomarkers, including a urine marker. We reasoned that a purely blood biomarker-based score would be of interest due to straightforward data collection in the clinical practice. On the other hand, a model considering all data may demonstrate superior performance.

##### Step 2: Validation of results

We assessed the performance of the derived risk score for CKD fast progressors in two subpopulations of the GCKD: in a first analysis (Subset 1), our objective was to select a subset of patients excluding those with late-stage CKD. The rationale for this is that we need to be able to observe disease progression - which is not possible if patients are already in late stages of disease. Therefore, we selected patients in Subset 1 with an eGFR measurement at baseline larger than $25 ml/min/1,73m^2$. In a second analysis (Subset 2), we wanted to mimic the diabetic kidney disease population of FIDELITY as closely as possible. For Subset 2, we selected patients with an eGFR larger than $25 ml/min/1,73m^2$ and in addition, only focused on those with a diagnosis of diabetes at baseline. As the time-to-event outcome, we used the first occurrence of either time to permanent kidney dialysis or time to kidney replacement.    

We estimated the risk score as outlined above and calculated the concordance index  [17] for both Subset 1 and Subset 2 by comparing the observed order of events with the risk score.

##### Step 3: Genetic analyses

We calculated the risk score, as described above, on the full quality-controlled set of UKBB participants. We provide an in-depth description of phenotyping, genotyping, our custom variant annotation pipeline, and statistical association testing in the Supplement.

In short, all genetic analyses were performed using the REGENIE statistical genetics analysis platform  [18]. Broadly, we performed two classes of genotype-phenotype statistical association testing: a) variant level analyses, in which all TOPMED-imputed  [19] germline genomic variants genome-wide were tested for phenotypic association and b) burden (or’collapsing’) gene-level tests (based on whole-exome sequencing data,  [20]), in which predicted deleterious coding Loss-of-function (LOF) and likely pathogenic missense variants were grouped and tested for phenotypic association on a per-gene basis, to increase statistical power. For the burden tests, variants were annotated in a number of different classes (‘masks’) based on computationally predicted pathogenicity using the Google AlphaMissense deep learning annotation algorithm  [21], and each per-gene mask was tested for phenotypic association. Further details on the analytical design, variant annotation, gene annotation and pathogenicity prediction methods are provided in the Supplementary Material.

We post-processed the output of our genetic analyses as follows. First, to facilitate interpretation of the GWAS results, we adjusted the p-values for genomic inflation calculated in genomic regions that did not reach genome-wide significance ($P < 5 \cdot 10^{-8}$). We started by defining significant loci, by extracting all variants that achieved genome-wide significance and extended the analysis to include 100 kb regions upstream and downstream. If multiple significant variants were found within this flanking region, the locus was extended until no additional significant variants were identified within 100 kb downstream. Next, we focused on variants not located within significant loci, to calculate an inflation factor (*λ*) using a chi-square statistic and the 1st percentile  [22]. If *λ* exceeded 1, we adjusted the p-values of all variants located in both significant loci and non-significant regions. This adjustment was performed by dividing the Z statistic *z* for each variant (calculated as the effect size *β* divided by the standard error $se(\beta)$) by *λ*, that is,

4 $$z*=\frac{z}{\lambda}=\frac{\beta/{se(\beta)}}{\lambda}. $$

The adjusted P-values are then derived based on the adjusted Z statistics $z^*$. After adjustment for genomic inflation, we defined significant loci using the same procedure as above using the adjusted P-values.

For the gene-based collapsing tests, we applied a Bonferroni correction and considered both the number of genes tested and the number of phenotypes tested, leading to the definition of a hit if $P < 2.8 \cdot 10^{-6}$ for a gene. Additionally, we filtered out any significant hits which were supported by $\leq 20$ heterozygous carriers, and any hits reported as significant but obtained via burden masks which only tested exceedingly rare variants (MAF $\leq 0.001$) or’singleton’ variants (further details in the Supplement).

SNP-based heritability (*h*^2^) and genetic correlations (*r*_*g*_) were estimated using LD Score regression as implemented in the LDSC  [23, 24] package. Summary statistics were processed with munge_sumstats.py using default settings, restricted to high-quality HapMap3 variants (INFO ≥ 0.9). The extended major histocompatibility complex (MHC/HLA) region on chromosome 6 (25–34 Mb) was excluded due to its complex linkage disequilibrium structure. Univariate LD Score regression was applied to estimate SNP-based heritability for each trait, and cross-trait LD Score regression was used to compute pairwise genetic correlations. All analyses used precomputed European LD scores and regression weights based on the GRCh38 reference panel.

##### Step 4: From genetic association testing to novel target candidates

It is essential to note that the focus of this work is not to reconfirm loci that were already reported as, for example, albumin-associated in the UKBB. Our objective was rather to attempt to identify novel loci, which would have been missed when performing straightforward GWAS analyses of each contributing kidney biomarker individually. Therefore, we systematically excluded ‘known’ hits, intended as genes or genomic loci that would have been obtained when performing identically specified GWAS analyses for each one of the constituent biomarkers of our CKD risk score.

For the SNP-based tests, we performed colocalization (coloc package  [25]) for all significant loci against the GWASs for the individual biomarker score components reaching genome-wide significance in the respective locus. We then excluded all loci with a value $H_4 > 0.5$ for any of the biomarkers. We mapped the identified genetic regions to the most plausible causal gene using the CALDERA  [26] package. CALDERA outputs a predicted causal probability (a value between 0 and 1). We prioritized genes that had a causal probability greater than 50% and had the highest score in their locus and excluded loci with no gene with a causal probability greater than 50%.

For the gene burden (collapsing) tests, we performed one association analysis against the CKD score, and one association analysis against each of the score biomarkers taken individually, and used custom scripts to intersect the Bonferroni-corrected lists of genes we generated. Any significant gene hit that was not ŉovel’ to the GCKD score-specific association test (e.i. was obtained via at least one of the ancillary burden tests performed against the score’s biomarker) was discarded from the final ranked list.

#### Computational details

All analyses were performed in R 4.0.1 [27] or Python 3.13.5  [28] or above. The packages tidyverse, survival, otargen, dplyr, qqman, GWASLab, LDSC and ggplot2 [7, 23, 24, 29–34] were used for the analysis.

## Results

In this Section, we demonstrate the application of the proposed framework for our case study.

### Risk score derivation

In FIDELITY, 41 biomarkers were measured at baseline and are available for the majority of subjects. These were included in our analysis. Of the 41 available, 19 were not measured in the UKBB and/or GCKD and were excluded, leaving us with 22 biomarkers for consideration for the urine model. For the non-urine model, in addition, two variables were removed (also measured in urine), leaving a candidate set of 20 biomarkers. Of those, 14 biomarkers for the urine and 12 biomarkers for the non-urine model were selected in the first stage of the variable selection procedure. In addition to the selected biomarkers, we considered age and sex for both models. All details on available markers and the first step of the selection procedure are outlined in the Supplementary material.

Table 1 shows the selected variables after multivariate modelling for the risk score. The models consist of 7 biomarkers (urine) and 8 biomarkers (non-urine) and include established kidney disease risk markers such as UACR and hemoglobin  [35]. There is a large overlap in terms of contributing biomarkers between the urine model and the non-urine model. We note that the directionality of effect differs for LDL cholesterol for the two models. This can be explained by the fact that single-model coefficients may not be interpretable due to the complex correlation structure between the biomarkers.

**Table 1:** The values represent the coefficients in the risk score. “-” indicates that the variable is not part of the respective model. BP: blood pressure, LDL Ch.: low density lipoprotein cholesterol, UACR: urinary urea creatinine ratio, BMI: body mass index. (**s**) serum

Trait	Urine model	Non-urine model
Systolic BP	0.18067	0.24191
LDL Ch.	0.01840	−0.33248
UACR	0.51748	-
Hemoglobin	−0.33272	−0.32613
Albumin (s)	−0.23264	−0.50993
BMI	−0.13045	−0.12958
Creatinine (s)	0.20419	0.26411
Cholesterol	-	0.43401
Age	-	−0.12259

Overview on selected variables for urine and non-urine data

Performance was assessed in the test data (internal validation): a concordance index of 0.8089 in the urine model and 0.7633 in the non-urine model indicated good separation between high-risk and non high-risk populations by the models.

In the Supplementary Material, we show how a reduction of biomarkers to the ones available in all three dataset does not decrease the prediction performance of the model (concordance index 0.8068 if we use all variables; see the supplementary analysis).

### Validation results

We used the GCKD biobank data to validate the derived models. After exclusion of patients with late-stage CKD ($eGFR\le 25ml/min/1,73^2$), 5,050 patients in the general population of CKD (diabetic and nondiabetic) and 1,793 patients in the diabetic CKD population were available for analysis. During follow-up, 152 of the 1,793 diabetic CKD patients and 382 of 5,050 diabetic and non-diabetic CKD patients reached the composite kidney endpoint.

As is the case for the test data set from the FIDELITY dataset, we observe an excellent performance of the model in discriminating between different risk levels of disease progression (Table 2). We note that performance is comparable to performance on FIDELITY and that there is no systematic difference in performance between diabetic CKD and non-diabetic CKD patients.

**Table 2:** Overview on model performance in validation study. Displayed is the concordance index for the respective setting

	Urine model	Non-urine model
Diabetic CKD	0.805	0.771
General CKD	0.802	0.774

### Genetic results

**Fig. 2:**
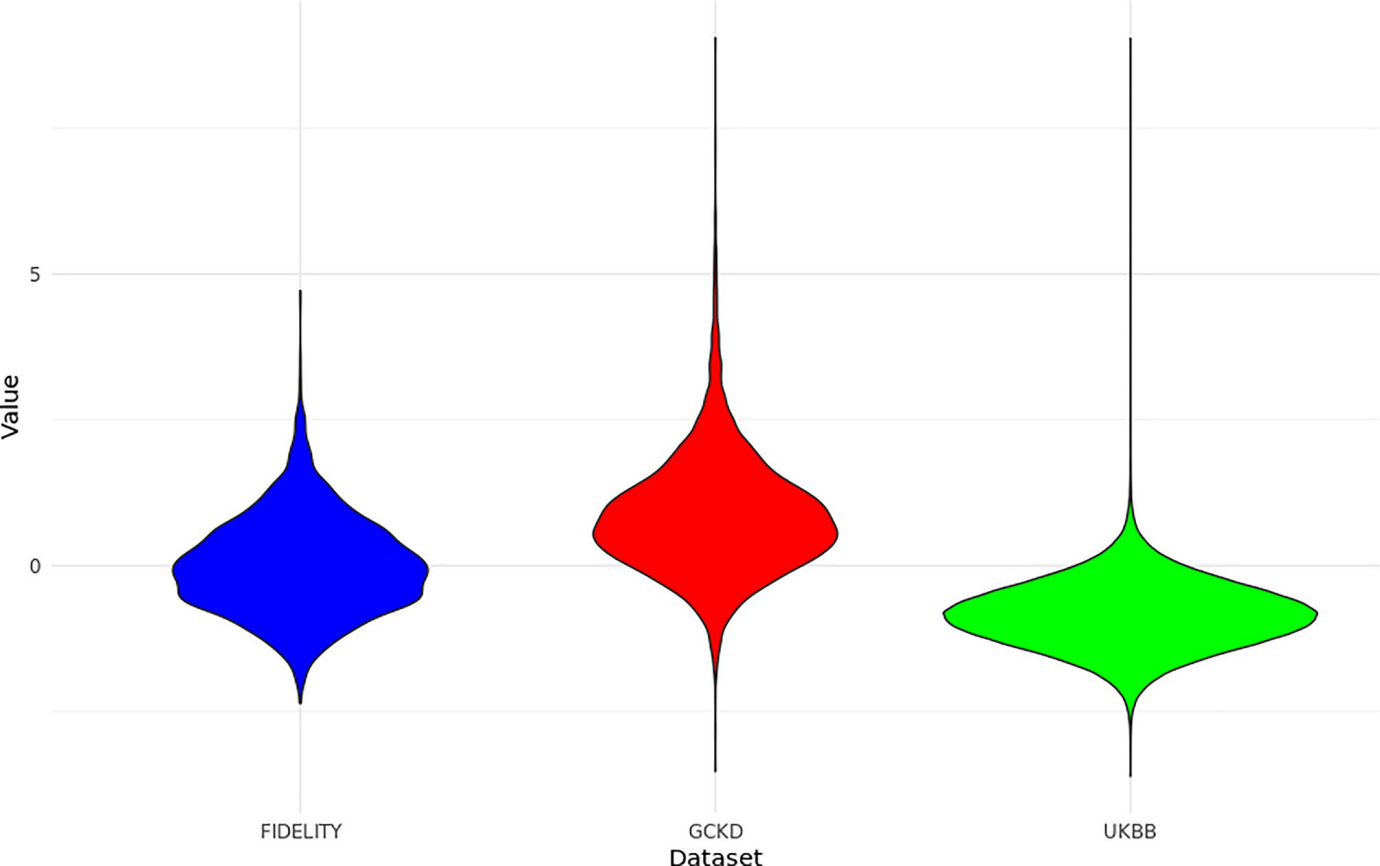
Distribution of the risk score without urine measurements across the three data sources

As a final step in the workflow, we transferred the two risk scores to the UKBB. The score and genetic data are available for 295,495 subjects (non-urine score) and for 287,422 subjects (urine score). The distribution of risk scores differed from that in FIDELITY and GCKD: there are more subjects with a low risk of rapid progression of CKD (Fig. 2 for the non-urine risk score). This is expected, given we included all UKBB participants for which our risk score could be built, and not all of these were diagnosed with kidney disease.

**Fig. 3:**
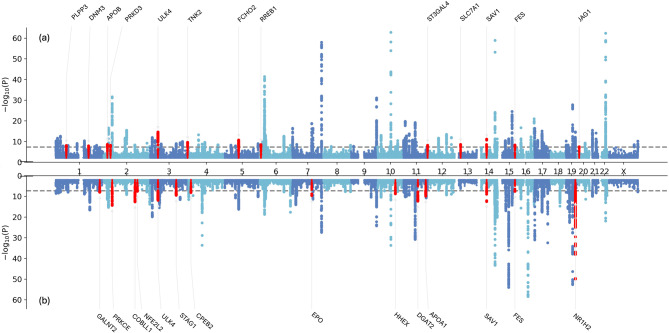
Miami plots for urine (**a**) and non-urine (**b**) score (corrected for genetic inflation). The y-axis for the non-urine score was truncated to align between urine and non-urine score. The red marks indicate hits that passed the novelity filter

For the SNP-based analysis, we obtained 123 hits for the non-urine score (120 hits for the urine score) (Fig. 3). Most plausible causal genes of 53 genetic regions are overlapping.

Genetic heritability, calculated from SNP-based analysis, is similar for urine ($h^2 = 0.0884 \pm 0.0045$) and non-urine score ($h^2 = 0.0839 \pm 0.0045$).

**Fig. 4:**
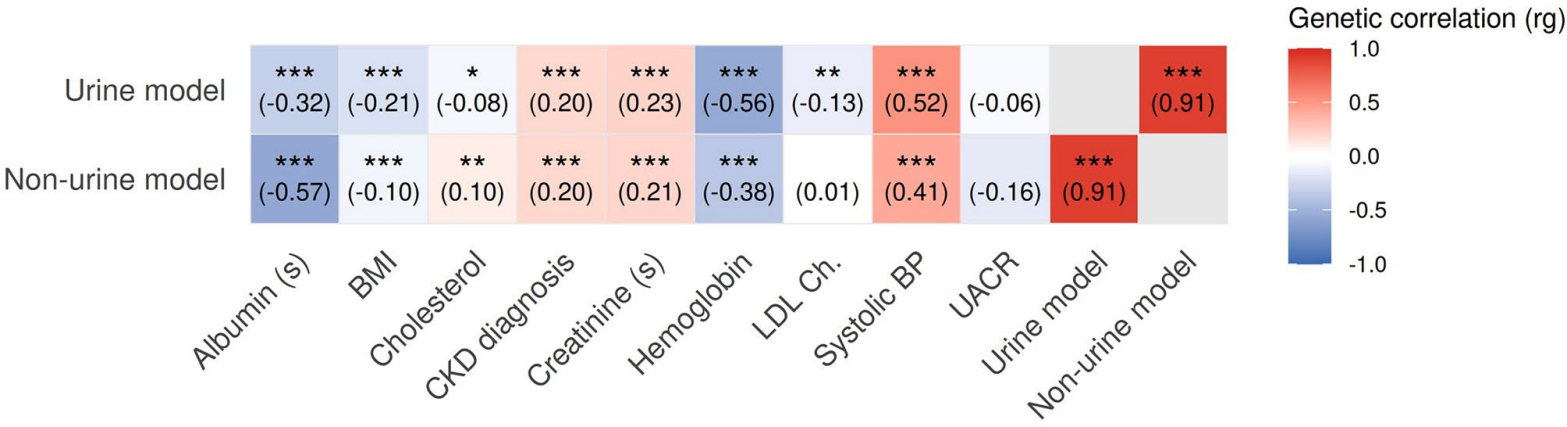
Genetic correlation between the two risk scores and to the score components. CKD diagnosis is a GWAS for CKD onset calculated in UK biobank (see supplement for definition of phenotype). Asterisks indicate genetic correlations that are statistically significant after Bonferroni correction. Significance levels: * $p < 0.05$, ** $p < 0.01$, *** $p < 0.001$

The genetic correlations between the traits are summarized in Fig. 4. We note that the genetic correlation between the two endpoints is highest ($r_g=0.91$) whereas the genetic correlation to the score component is lower, absolute values ranging between $r_g=0.06$ (UACR vs. urine score) and $r_g=0.57$ (albumin and non-urine score).

Of the identified regions, 25 (urine score) and 20 (non-urine score) met our definition of novelty (i.e., no co-localization of genome wide significant hit to any score component). For the urine score, 13 of those could be mapped with confidence (causal probability of CALDERA $\ge 0.5$). For the non-urine score, this is true for 14 loci. For the gene-based tests, we obtained 25 hits for the non-urine score and 13 for the urine score, of which 3 hits for the non-urine score and 2 hits for the urine score satisfied our definition of novelty (no overlapping hit below significance threshold for any other score component). Note that 2 of these genes are overlapping. A visual overview of hits for the gene-collapsing test is displayed in the Supplementary Material.

Table 3 gives an overview of all prioritized genetic regions. A list with all identified regions, as well as plausible candidate genes can be found in the Supplement (’locus_overview.xlsx’).

**Table 3:** ’Urine’ = score with urine measurements, ’No urine’ = score without urine measurements. ’Both’ = hit detected via both scores. Mapped gene refers to the gene with highest CALDERA score. We only display genetic regions with a CALDERA score larger than 0.5. For the burden test hits, reported *p*-value is the smallest multiple-hypothesis corrected *p*-value observed across all tests performed on the gene

GWAS/Burden	SNP/Gene	P	Score	Mapped gene	CALDERA score
GWAS	1_230157783_C_T	9.64701e-09	No urine	GALNT2	0.86
GWAS	2_46138781_G_A	4.05034e-15	No urine	PRKCE	0.97
GWAS	2_164692059_G_A	6.1629e-14	No urine	COBLL1	0.96
GWAS	2_177127422_C_T	2.73617e-08	No urine	NFE2L2	0.60
GWAS	3_41983035_A_G	1.16655e-12	No urine	ULK4	0.66
GWAS	3_136715991_A_C	3.08731e-10	No urine	STAG1	0.52
GWAS	4_14958378_T_G	1.06282e-08	No urine	CPEB2	0.85
GWAS	7_100722010_G_A	2.17194e-10	No urine	EPO	0.98
GWAS	10_92672243_C_T	1.63487e-09	No urine	HHEX	0.69
GWAS	11_75828689_A_G	4.40856e-13	No urine	DGAT2	0.82
GWAS	11_116901725_C_T	1.16397e-10	No urine	APOA1	0.71
GWAS	14_50666797_C_A	7.14863e-13	No urine	SAV1	0.74
GWAS	15_90887387_G_A	2.39493e-08	No urine	FES	0.66
GWAS	19_49521951_T_A	4.0567e-43	No urine	NR1H2	0.58
GWAS	1_56458696_G_GA	9.19674e-09	Urine	PLPP3	0.74
GWAS	1_172162145_C_T	1.34159e-08	Urine	DNM3	0.75
GWAS	2_21195211_G_A	2.46419e-09	Urine	APOB	0.73
GWAS	2_37290423_A_G	6.6238e-09	Urine	PRKD3	0.60
GWAS	3_42001222_C_T	2.76248e-15	Urine	ULK4	0.55
GWAS	3_195908561_G_A	2.50119e-10	Urine	TNK2	0.54
GWAS	5_72825297_A_G	1.96801e-11	Urine	FCHO2	0.75
GWAS	6_7211585_G_A	3.27023e-09	Urine	RREB1	0.78
GWAS	11_126407675_A_G	6.68468e-09	Urine	ST3GAL4	0.69
GWAS	13_29563691_T_C	2.78347e-09	Urine	SLC7A1	0.79
GWAS	14_50666797_C_A	2.16961e-11	Urine	SAV1	0.61
GWAS	15_90887387_G_A	5.03789e-09	Urine	FES	0.54
GWAS	20_10688540_C_A	3.67708e-08	Urine	JAG1	0.76
Burden	COL4A3	5.65185e-09	No Urine	-	-
Burden	FNIP1	8.35313e-09	Both	-	-
Burden	TTK	3.16896e-08	Both	-	-

Identified genetic regions for variant-level testing (SNP) and gene-level testing (burden)

As expected, some of the genetic regions on the initial hit list (before novelty filtering) are known loci for end-stage renal disease or kidney biomarkers (e.g., albumin, eGFR, UACR). For example, we identified a region with the lead SNP rs1260326 (2_27508073_T_C) that has been reported to be associated with an increased risk of end-stage renal disease in patients with Type 2 diabetes  [36]. This region colocalizes in our analysis with albumin, cholesterol, creatinine, hemoglobin, and LDL and was therefore excluded as part of the novelty screening. After novelty check, none of the regions identified in both the SNP-based analysis and the gene-based analysis are listed as a hit in AZPhewas  [37] for the ICD code N18 (Chronic kidney disease) - suggesting that a simple GWAS for CKD would not have produced the same result.

Although none of the regions were reported to be genetically associated with CKD, a manual PubMed search  [38] of the identified loci showed that some of them had already been described in the broader context of kidney diseases. For example, *COL4A3* in the context of Alport disease nephropathy  [39] or *PRKCE* for kidney injury response. Others, like *DNM3* and *TNK2*, have, to the best of our knowledge, not previously been described in the context of kidney disease. Their role in the progression of kidney disease and their potential as drug target candidates have yet to be established.

## Discussion

In this paper, we proposed a’back-translation’ approach to target hypothesis discovery, and presented a framework for integrating clinical and biobank data to advance the mechanistic understanding of disease and to facilitate the identification of potential new drug targets. We demonstrated the applicability of the framework in practice by designing a case study for a subpopulation of chronic kidney disease (CKD) fast progressors. We defined this based on the placebo arm of two clinical studies that investigated Finerenone, we performed validation in the GCKD cohort and used statistical genetics approaches to variant- or gene-phenotype association in the UKBB to pin down its likely molecular signifiers. Our approach to identify novel genetic signals associated with the progression of CKD disease identified multiple loci through the analysis of common genetic variation and through gene-based burden testing. A plausibility analysis shows that the framework is able to find mechanistically relevant genes and potential targets for fast progressors of CKD. We emphasize that the back-translation approach can in principle be used for any phenotype/indication of interest as long as detailed clinical data are available to derive a well supported risk score or prediction model.

The prediction ability of the derived risk score in our sample use case is excellent in both internal and external validation studies. Validation is crucial and generates confidence that the risk score constructed based on clinical trial data is also meaningful in biobank data. We were unable to perform a validation of the score directly within the UKBB due to the scarcity of data points and to missing longitudinal data collection, which hampered interpretation. Genetic analyses identified a number of associated genomic regions. We hypothesize that these contain genes involved in mechanisms of CKD progression and are likely to include interesting target hypotheses for follow up. We also showed that our proposed framework is capable of identifying genetic regions that would not have been picked by running straightforward association testing against any one of the participating diagnostic labels in the UKBB taken individually. Although none of our novel classified regions has been previously reported to have a direct genetic association with CKD, several identified genes had been flagged by existing studies in relation to kidney disease in general.

COL4A3 is one of three known collagen 4 genes responsible for Alport syndrome  [39], a genetic disease which affects the kidneys, also causing loss of hearing and eye abnormalities. Pathogenic variants causing Alport syndrome also occur frequently in patients with other kidney diseases, for instance focal and segmental glomerulonephritis or familial immunoglobulin A  [40]. Our work points to a possibly even broader role extending to Chronic Kidney Disease.

FNIP1 has been described previously in the context of the Birt–Hogg–Dube syndrome, an autosomal dominant inherited disease which can lead to lung cysts and an increased risk for renal tumors. FNIP1 is an interactor of the encoded protein of the causal gene  [41]. Recently, experimental evidence was published on the role of FNIP1 in the context of polycystic kidney disease  [42]. Determining the role of FNIP1 in rapid CKD progression may be a field of future research.

We note that the approach relies on the identifiability of a subgroup of interest within the available biomarker data. Although this appears to be the case for our case study, it may not be true for all indications, which may limit the applicability of our approach to a broader context. In addition, the ability to validate the identified risk score using an external biobank is crucial to increasing confidence in back-translation to biobank data. However, external validation via a second biobank is not always feasible. When investigating non-responders to a drug currently in development, the absence of relevant real-world data renders external validation impractical. In such cases, researchers must rely on a model without the benefit of validation, which can undermine the robustness of the findings. However, the ’back-translation’ approach is specifically appealing in these situations: in these cases, there is no data at all available in the biobank rendering the need for bridging between different data sources essential. It should be noted that in our case study we could have also conducted a validation in the clinical data by analyzing FIDELIO-DKD and FIGARO-DKD separately and using one study for model building and one for validation. This may be an attractive alternative to a fully external validation.

An additional limitation of this study is the interpretation of the risk score for CKD fast progression within a general, partially healthy population, raising questions about the utility of genetic information in this context. Restricting the study population in UKBB to a disease-matched population in line with the FIDELITY dataset is not straightforward for several reasons: first, if we require a high similarity between the two study populations, we severely reduce the sample size for the genetic assessment. This leads to low power and may result in a lack of genetic signal. Furthermore, the interpretation of results, generating after restricting to a disease cohort, is challenging due to collider biases  [43]. An in-depth evaluation on how population-matching can be applied in this context is currently ongoing as a methodological research theme in our team.

Using the population-wide approach presented in this manuscript, we aim to establish the plausibility of results by presenting positive controls. For that, we use the gene list prior to the novelty filtering (restricted to those with high confidence in the identification of the causal gene, i.e., CALDERA ≥ 0.5) and note that several are currently or have been previously studied in kidney populations. For example, ABAC1 is a drug currently studied in Phase II in patients with diabetes and kidney disease [44]. Outcomes are expected in 2026. We also identify TGF-*β*  [45] and IL6  [46], two known drug targets which have been explored in kidney indications. However, although at least some of the identified genetic regions have been reported in the literature, suggesting validity of the approach, the quality of these regions as drug target candidates for “CKD fast progressors” remains uncertain. Consequently, more research is essential to determine whether these targets can advance through subsequent stages of drug R&D. In addition, the challenge of effectively developing targets for fast progressors of CKD persists, particularly with respect to the possible use of the risk score to accurately identify the relevant patient population.

The framework we propose addresses a significant challenge that stems from the lack of genotyping in clinical trials, which hinders our ability to effectively study the mechanisms underlying identified subgroups. If genotyping were conducted routinely in large clinical trials, researchers could directly investigate these mechanisms, facilitating a more straightforward analysis. This would ultimately improve the value of these trials as the generated data could routinely be used for research efforts even after the investigated treatment becomes available to all patients. Furthermore, integrating other omics layers could improve translation between clinical trials and biobank data, as omics is widely regarded to be more closely aligned with the underlying mechanisms of disease than traditional phenotyping approaches.

## Conclusion

We propose a novel framework for data-driven target identification for the CKD fast progression indication, based on the combined usage of clinical and genetic data. The framework can be applied to multiple therapeutic areas. It offers a distinctive opportunity to incorporate clinical data into the target identification process through “back-translation”. Using previously overlooked clinical insights, our framework connects clinical and genetic data, tracing a new path towards uncovering novel therapeutic target candidates, which does not rely on literature data or custom knowledge base-derived search approaches. This purely patient-data driven, integrated strategy marks a significant advancement in the search for more effective personalized treatment options in healthcare.

## Electronic supplementary material

Below is the link to the electronic supplementary material.

Supplementary material 1: Supplementary methods

Supplementary material 2: Variable overview

## Data Availability

All data generated or analysed during this study are included in this published article and its supplementary information files. Summary statistics for genetic analysis are deposited in the GWAS catalogue. Individual-level data from the UKBB can be obtained from the UKBB with an approved research proposal. FIDELIO-DKD and FIGARO-DKD data are accessible via https://vivli.org/.

## Abbreviations

BL: Baseline.
BMI: Body Mass Index.
SNP: Single Nucleotide Polymorphism.
CKD: Chronic Kidney Disease.
GCKD: German Chronic Kidney Cohort.
GWAS: Genome-wide association studies.
T2D: Type 2 diabetes.
UACR: Urine albumin-creatinine ratio.
eGFR: Estimated glomerular filtration rate.
LDL: Low Density Lipoprotein.
BP: Blood Pressure.
ICD: International Classification of Disease.
UKBB: UK Biobank.
