# Supplementary material for: An integrative framework for drug target discovery bridging clinical trial and genetic data insights
Source: J Transl Med. 2026 Mar 13;24:374. doi: 10.1186/s12967-026-07806-x (PMC12994227; doi:10.1186/s12967-026-07806-x)
Supplement: Supplementary file 1 — Supplementary material 1: Supplementary methods [file 12967_2026_7806_MOESM1_ESM.pdf]

Supplementary material — An integrative framework for drug target discovery bridging clinical insights and genetic data

Contents

1 Supplementary methods **1**
1.1 Data and data pre-processing 1
1.1.1 FIDELITY dataset 1
1.1.2 GCKD 2
1.1.3 UK Biobank 3
1.1.4 Scaling and alignment of units 4
1.2 Genotype-Phenotype Association Analyses in the UKBB 4
1.3 Statistical genetic association testing framework — the Regenie Pipeline 6
1.4 Definition of a CKD phenotype in UK Biobank 7

2 Additional analyses **8**
2.1 Sensitivity Analysis 8

3 Supplementary figures **9**

1 Supplementary methods

1.1 Data and data pre-processing

1.1.1 FIDELITY dataset

The FIDELITY dataset has been a pre-defined, exploratory pooled analysis of individual patient data from two Phase III trials, FIDELIO-DKD Bakris et al. (2020) and FIGARO-DKD Pitt et al. (2021), and has been discussed in detail before (Agarwal et al., 2022).

Inclusion criteria Both FIDELIO-DKD and FIGARO-DKD were international, randomized, double-blind, placebo-controlled, multicenter trials with complementary objectives. The design, baseline characteristics, eligibility criteria, and primary findings of these studies have been previously published. In summary, all participants provided written informed consent, and the studies were conducted in accordance with the principles of the Declaration of Helsinki, receiving approval from an ethics committee at each participating site. Adult patients with type 2 diabetes (T2D) and chronic kidney disease (CKD) were enrolled, defined by an estimated glomerular filtration rate (eGFR) of $25 - 90$ mL/min/1.73 m^2 and a urine albumin-to-creatinine ratio (UACR) of $30 - 300$ mg/g, or an eGFR of ≥ 25 mL/min/1.73 m^2 with a UACR of $300 - 500$ mg/g. Participants were required to be on the maximum tolerated labeled dose of an ACE inhibitor or angiotensin receptor blocker for at least four weeks prior to screening. Key exclusion criteria included: (i) heart failure with reduced ejection fraction and New York Heart Association Classes II–IV (Class 1A recommendation for mineralocorticoid receptor antagonists); (ii) significant known non-diabetic kidney disease; (iii) occurrence of stroke, transient ischemic attack, acute coronary syndrome, or hospitalization for worsening heart failure within 30 days prior to the screening visit; (iv) dialysis for acute renal failure within 12 weeks before the run-in visit; and (v) any other condition that would render the patient unsuitable for the study and impede participation for the entire planned study duration.

Definition of relevant endpoints and pre-processing of biomarkers We use the primary endpoint of the FIDELIO-DKD study which is a composite outcome including kidney failure, a sustained decrease of at least 40% in the eGFR from baseline, or death from renal causes (Bakris et al., 2020)

For the risk score construction, we use all available biomarker measurement at baseline which are overlapping with biomarker data available in UKBB (see Supplementary table 'variable_overview.xlsx'). Since only few biomarker measurements were missing, missing data was imputed with a median imputation. Visual inspection of distributions was conducted and it was decided not to do any additional data cleaning and/or pre-processing.

1.1.2 GCKD

The GCKD has been described in detail before (Eckardt et al., 2012). Here, we focus only on the most relevant aspects for our study.

Inclusion criteria and study set-up In summary, the inclusion criteria for the study were participants aged between 18 and 74 years who met either of the following conditions: a) an estimated Glomerular Filtration Rate (eGFR) of $\leq$ 60 ml/min/1.73m^2, or b) an eGFR $\geq$ 60 ml/min/1.73m^2 along with albuminuria greater than 300 mg/g creatinine or proteinuria exceeding 500 mg/g creatinine. The exclusion criteria included individuals of non-Caucasian descent, those with a history of solid organ or bone marrow transplantation, individuals diagnosed with an active malignancy within 24 months prior to screening, those suffering from Heart Failure Class IV as classified by the New York Heart Association (NYHA), and individuals who were under legal guardianship or unwilling to provide consent.

At baseline (BL), a comprehensive phenotyping of study participants was conducted, which included gathering details on medical history, prescription medications, and responses to questionnaires about health-related factors and symptoms associated with cardiovascular disease (1). Additionally, clinical measurements were taken at baseline, such as blood pressure readings, and certified study nurses collected plasma, serum, and spot-urine samples at regional GCKD centers or nephrology practices of participating doctors. The collected samples were then processed, frozen, and sent to a central biobank for storage and future analysis. A range of laboratory parameters was assessed by a central laboratory prior to the samples being stored. Longitudinal data regarding the participants' health status, including hospitalizations, was collected through regular phone interviews and during follow-up assessments at 2, 4, and 6 years.

Definition of relevant endpoints and pre-processing of biomarkers Table 1 gives an overview on the biomarkers in GCKD. We use the BL assessment as time point. Subjects with missing data were excluded from the analysis. After visual inspection, it was decided not to perform additional systematic data cleaning.

Table 1: Overview of definition of biomarkers in GCKD. s = serum.

Trait	Variable name
UACR	uacr_bl
Hemoglobin	hemovalue_bl
Albumin (s)	albuvalue_bl
BMI	bmi_korr_bl
Age	age_bl
Systolic BP	bloodpr_sys_bl
Cholesterol	cholvalue_bl
LDL Ch.	ldlvalue_bl

For the validation of the model, it is necessary to define a kidney composite endpoint which is as similar to the one used in FIDELITY as possible. Here, we define the event as either permanent

dialysis or kidney replacement therapy.

We run the validation analysis also in a diabetic CKD subpopulation which is closer aligned to the FIDELITY population than a general CKD population. We define as a diabetic CKD anyone with a positive entry for diabetes at baseline.

1.1.3 UK Biobank

The UK Biobank has been described in detail before (Bycroft et al., 2018) and we thus focus only on the most relevant aspects.

Inclusion criteria and study set-up The UK Biobank is based on voluntery participation of individuals aged 40-69 at enrollment. Exclusion criteria included individuals with certain medical conditions or disabilities that would prevent them from fully participating in the baseline assessment. The baseline assessment itself was comprehensive, involving the collection of extensive health and lifestyle information, physical measurements, and biological samples from each participant.

Pre-processing of phenotypes The selected quantitative traits are outlined in Table 2. We use the baseline assessment (Instance 0). Most of these traits are directly sourced from a specific UK Biobank field, as indicated in the table under the "Field" column. Other traits are derived from a combination of field data, with the calculation process detailed in the "Details" column.

To eliminate extreme outliers, implausible values, and to minimize data skewness, the quantitative traits underwent systematic cleaning. The choice of method for each phenotype was made following a visual inspection. The approaches used, in the specified order, are described in the "QC" column in Table 2.

- Exclusion of isolated outliers (labeled as "outlier" in Table 2): A step-wise procedure was employed. Initially, the median absolute deviation (MAD) was calculated, and for each observation (x), we checked how many other observations (from different subjects) fell within the range If fewer than 30 other observations were found in this interval, the observation was deemed isolated and its value was set to NA.
- Percentage deletion (labeled as "percentage" in Table 1): This involved removing the top and bottom 0.1% of ordered observations.

Subjects with missing data were excluded from the analysis.

Table 2: Overview of definition of biomarkers in UK Biobank. s = serum, u = urine.

Trait	Field	Detail	QC
UACR	30500/30510	Ratio albumin/creatinine (urine)	-
Hemoglobin	30020	-	percentage, outliers
Albumin (s)	30600	-	outliers
Creatinine (s)	30700	-	-
BMI	23104	-	outliers
Age	21003	-	-
Systolic BP	4080	-	outliers
Cholesterol	30690	-	-
LDL Ch.	30780	-	-

In line with other publications, we imputed UACR values below the lower level of detection by setting it to the lower level of detection (Casanova et al., 2019).

1.1.4 Scaling and alignment of units of measurement

To allow for translation of models between data sources, it is important to align units of measurements. Here, all units of measurements are aligned to the FIDELITY dataset as outlined in Table 3.

In addition, all selected biomarkers are normalized with the mean and the standard deviation from the FIDELITY study. This ensures that the relationship between the coefficients of the risk score is preserved across datasets.

Table 3: Overview of selected traits and units of measurements. s = serum, u = urine, U = Unit, T = Transformation. For the Transformation, we give the factor with which we multiply the measurements in the respective datasets.

Trait	U FIDELITY	U UKBB	T UKBB	U GCKD	T GCKD
UACR	-	-	-	-	-
Hemoglobin	g/dL	g/dL	-	g/dL	-
Albumin (s)	g/dL	g/L	0.1	g/L	0.1
Creatinine (s)	mg/dL	umol/L	0.01	mg/dL	-
BMI	kg/m^2	kg/m^2	-	kg/m^2	-
Age	years	years	-	years	-
Systolic BP	mmHg	mmHg	-	mmHg	-
Cholesterol	mg/dL	mmol/L	38.67	mg/dL	-
LDL Ch.	mg/dL	mmol/L	38.67	mg/dL	-

1.2 Genotype-Phenotype Association Analyses in the UKBB

We used the genetic data in the UKBB to identify variants or genes showing statistically significant associations with the CKD risk score and individually with the score's constituent biomarkers, as described in the main manuscript. Broadly, we performed two classes of analysis:

1. **Exome-wide, coding variant collapsing (burden) tests** — we focused on highly curated lists of variants called in the Whole Exome Sequencing (WES) set of 470K individuals in the UKBB. After QC and filtering to retain only data from consenting individuals of European origin, we annotated the variant set for predicted deleteriousness and impact on protein product and, to increase statistical power, we grouped predicted deleterious and likely pathogenic variants in gene-level and minor-allele-frequency (MAF) thresholded subsets, and tested each per-gene set for associations with the CKD risk score. We also performed separate, identical tests against the CKD score constituent biomarkers individually;

2. **Genome-wide, common variant analyses** — we employed all UKBB genotype data from the SNP-Chip array set, restricting to the European ancestry of the cohort, and imputed as part of the Trans-Omics for Precision Medicine (TOPMed, (Taliun et al., 2021)) programme, to discover associations between small genomic variation genome-wide (including non-coding variants) and the CKD risk score or its constituent biomarkers.

In the next two paragraphs, we will further detail our methods to perform the above.

UKBB Whole Exome Sequencing Coding Variant Annotation

We used the Ensembl Variant Effect Predictor (VEP) V.111 (McLaren et al., 2016) to annotate all QC-ed coding variants in the 470K WES release of the UKBB. Variants that were not annotated as a `protein coding` or `lncRNA` VEP-labelled GENCODE construct were not considered for further analysis.

To obtain *in silico* predictions for the likely pathogenicity of the variants annotated by the VEP tool as 'missense' (given a chosen transcript) we used the Alpha Missense AI variant annotation algorithm (Cheng et al., 2023), which was run as a VEP plugin.

Additionally, to derive high quality annotations for stop-inducing, frameshift-inducing or alternative splice-inducing variation in the set (collectively referred to as 'loss-of-function' or LOF predicted deleterious variants) we ran the GnomAD (Karczewski et al., 2020) project's Loss-Of-Function Transcript Effect Estimator (LOFTEE algorithm, `https://github.com/konradjk/loftee`), with standard options and once again as a VEP plugin.

The complete VEP command line we employed was the following:

```
vep --offline --cache -i ~{vep_in} ~{fork_arg} \
       --assembly GRCh38 --vcf \
       --everything \
       --show_ref_allele --hgvsg --humdiv \
       --overlaps \
       --dir_cache /opt/vep/.vep \
       --dir_plugins /opt/vep/.vep/Plugins/ \
       --plugin LoF,loftee_path:/opt/vep/.vep/Plugins/,\
                    conservation_file:/opt/vep/loftee_data/loftee.sql,
                    human_ancestor_fa:
                    /opt/vep/loftee_data/human_ancestor.fa \
       --plugin AlphaMissense,file=~{AlphaMissensehg38} \
       --output_file ~{base}.vep.vcf ~{extra_args}
```

Post-processing of the annotated variants To finalize a set of stringent likely pathogenic missense or predicted deleterious LOF variant predictions from the annotations produced with VEP, we further post-processed the VEP output to retain only variants labeled with consequence `missense+` (e.i. 'missense or worse'). We used the ranking provided by the Ensembl Consortium[1] for this purpose.

Importantly, to make sure we would not contaminate the data with variants predicted to be pathogenic on poorly annotated or biologically unsupported transcripts, we only retained variant consequences detected on the MANE transcript[2] (Matched Annotation from NCBI and EMBL-EBI) transcript for each gene. If the MANE transcript was not available for a given gene, we fell back on considering variant consequence on its canonical transcript.

We then defined two classes of relevant coding variant annotations to use for all our rare variant collapsing tests, as follows:

1. **Definition of likely pathogenic missense variant** — We labelled a missense variant annotated in a MANE transcript in any human protein coding or lncRNA gene as '`missense_am`' if the assigned alpha missense score $v_{\text{am}} > 0.564$, following the thresholds recommended in the original Alpha Missense publication.

2. **Definition of predicted deleterious high confidence LOF variant** — We labelled a LOF variant annotated by LofTee as '`LOF`' if the variant was labelled as 'LOF' and, simultaneously, if the LOF was not annotated with anomalous flags and if the LOF had been labelled as 'high confidence' by LOFTEE.

We used the variants above to create several sets of per-gene likely pathogenic or predicted deleterious variants, and used them as an input for our collapsing tests. We call these per-gene collapsing models 'masks'.

[1]`https://useast.ensembl.org/info/genome/variation/prediction/predicted_data.html`
[2]`https://www.ncbi.nlm.nih.gov/refseq/MANE/`

1.3 Statistical genetic association testing framework — the Regenie Pipeline

We used the Regenie statistical genetics testing framework (Mbatchou et al., 2021) for all our tests aiming to identify genetic associations to our backtranslated risk score.

The Regenie pipeline was run using the developer's recommendations for a Regenie UKBB analysis[3]. Briefly, the UKBB non imputed SNP-Chip data was aggregated and filtered to build the polygenic risk score used by Regenie as an association prior for the actual association testing in Phase II.

The following Plink command was used to pre-filter the common SNPs aggregate from the UKBB per-chromosome calls:

```
plink2 \
   --bfile  \
   --chr 1-22, X, Y, XY \
   --keep ~{qc_fam} \
   --output-chr MT \
   --maf 0.01 --mac 100 --geno 0.1 --hwe 1e-15 \
   --mind 0.1 \
   --indep-pairwise 1000 100 0.9 \
```

For Phases I and II of the pipeline, standard options were used, unless specified in the two subsections below which provide additional detail on the two main classes of analyses we performed.

For all analyses, the CKD risk score was reformatted to fit Regenie's input requirements, and a custom covariate file (age, sex, array_type, first 10 PCA eigenvalues) was used throughout.

Rare Coding Variant Collapsing Tests setup

For the rare coding variant testing, we created genome-wide gene-level annotation using the likely pathogenic and predicted deleterious variants described before.

There is a precision/recall trade-off when annotating variant pathogenicity *in silico*: on the one hand, using a small number of extremely well supported, high confidence, very rare variants (typically LOFs) might lead to small carrier numbers, resulting in limited power to detect associations; on the other hand, supplementing the annotated variant set with a large number of medium confidence variants (typically, likely pathogenic missense) will increase carrier numbers but will also reduce precision and introduce noise.

In order to provide a good balance between sensitivity and specificity of the range of genotyping annotation employed for our tests, we followed REGENIE's guidelines and created several collections of annotated gene-level variant data known as 'masks'. For each gene, the following masks were created:

Mask1 Considers high confidence loss-of-function variants only

Mask2 Considers high confidence loss-of-function as well as missense variants flagged as 'likely pathogenic' by the AlphaMissense algorithm

Mask3 Considers missense variants flagged as 'likely pathogenic' only by the Alphamissense algorithm

Mask4 Considers high confidence loss-of-function variants as well as all variants labelled as 'missense' (disregarding pathogenicity labelling) by Ensembl VEP.

Variants for each gene were further stratified based on Minor Allele Frequency (MAF) in the population, and divided in 4 strata, each with MAF upper boundary $\leq$ [singleton, 0.001, 0.01, 0.05].

The above resulted in a total of 4 (annotation mask) $\times$ 4 (MAF mask) = 16 tests per gene.

[3] https://rgcgithub.github.io/regenie/recommendations/

Post-processing of the Exome-wide, coding variant burden tests

The summary statistics for the burden tests against the score and its constituent biomarkers (6 tests, $N = 17,771$ genes) were further processed in a number of ways. Association p-values were Bonferroni corrected (with threshold $p_{\text{Bonf}} = .05/N = 2.8E-6$); any significant tests supported by $N_C \leq 20$ cases were dropped; all masks obtained with an upper MAF threshold of class 'singleton' or MAF $\leq .001$ were dropped; only significant associations obtained via 'mask1' or 'mask2' tests were retained.

The resulting list of gene-level hits associated with the CKD score was intersected with the identically filtered lists of gene-level hits associated with each of the constituent score biomarkers, and any hits figuring in both the CKD score set and any one of the constituent biomarkers score sets were dropped from further analysis.

1.4 Definition of a CKD phenotype in UK Biobank

The CKD onset phenotype is defined based on ICD (hospital records, ICD9/10, OPCs) and Read codes (read2/read3 codes). In addition, information from the assessment center interviews are used. We define a participant as a CKD patient if any of the following code were recorded during the participation in UK Biobank.

Read2 Codes K05..; K050.; G222.; G233.; G234.; K051.; K052.; K053.; K054.; K055.; K0D..; Kyu21; 7B00.; 7B000; 7B001; 7B002; 7B003; 7B004; 7B005; 7B00y; 7B00z; 7L1A.; 7L1B.; 7L1C.; 7B006; 1Z1..; K0E..; 1Z1M.; 1Z1N.; 1Z1P.; 1Z1Q.; 1Z1R.; 1Z1S.; 1Z1a.; 1Z1b.; 1Z1c.; 1Z1d.; 1Z1e.; 1Z1f.; 1Z1T.; 1Z1V.; 1Z1W.; 1Z1X.; 1Z1Y.; 1Z1Z.; 1Z10.; 1Z11.; 1Z12.; 1Z13.; 1Z14.; 1Z15.; 1Z16.; 1Z17.; 1Z18.; 1Z19.; 1Z1A.; 1Z1B.; 1Z1C.; 1Z1D.; 1Z1E.; 1Z1F.; 1Z1G.; 1Z1H.; 1Z1J.; 1Z1K.; 1Z1L.; X30D2; X30In; X30Iz; X30J0; X30J1; X30J2; X30J3; Xa3x6; Xa3x7; Xa3x8; XaB9D; Xac9y; Xac9z; XacA2; XacA4; XacA6; XacA9; XacAb; XacAd; XacAe; XacAf; XacAh; XacAi; XacAM; XacAN; XacAO; XacAV; XacAW; XacAX; XaLHG; XaLHH; XaLHI; XaLHJ; XaLHK; XaM1o; XaM1p; XaMKM; XaNbn; XaNbo; XaO3p; XaO3q; XaO3r; XaO3s; XaO3t; XaO3u; XaO3v; XaO3w; XaO3x; XaO3y; XaO3z; XaO40; XaO41; XaO42; XE0df; XM19D; XM19E

Read3 Codes X30In; X30J0; X30J1; X30J2; X30J3; XE0df; G222.; G233.; G234.; K05..; Kyu21; X30Iz; XM19D; XM19E; XaB9D; XaLHG; XaLHH; XaLHI; XaLHJ; XaLHK; XaNbn; XaNbo; XaO3p; XaO3q; XaO3r; XaO3s; XaO3t; XaO3u; XaO3v; XaO3w; XaO3x; XaO3y; XaO3z; XaO40; XaO41; XaO42; Xac9y; Xac9z; XacA2; XacA4; XacA6; XacA9; XacAM; XacAN; XacAO; XacAV; XacAW; XacAX; XacAb; XacAd; XacAe; XacAf; XacAh; XacAi; 7B00.; 7B000; 7B001; 7B002; 7B00y; 7B00z; 7L1A.; X30D2; Xa3x6; Xa3x7; Xa3x8; XaM1o; XaM1p; XaMKM

ICD9 585; 5859; 4030; 4031; 4039; 4040; 4041; 4049

ICD10 I120; I131; I132; N18; N180; N181; N182; N183; N184; N185; N188; N189

OPCs M01; M011; M012; M013; M014; M015; M018; M019; X40; X41; X42; X401; X402; X403; X404; X405; X406; X407; X408; X409; X411; X412; X418; X419; X421; X428; X429

Self-reported data 1192; 1193; 1519; 1520; 1607; Field 20004 (Code 1195)

2 Additional analyses

2.1 Sensitivity analysis: performance of a risk model in full set of biomarkers in the FIDELITY dataset

For the risk model that was derived in this paper, we restricted the set of candidate biomarkers to the ones available in the FIDELITY dataset, UKBB and GCKD. It is important to understand if this restriction limits the performance of the model. For that, we construct a risk model in the same way as described in the method section, but taking into account all 41 biomarkers available in the FIDELIITY dataset. We compare the model to the model with the restricted parameter set that includes the urine markers. Results are displayed in Table 4: we note that the models largely overlap (including the coefficients of the parameters). Only hematocrit and hemoglobin are changed; however, it is well-known that these are highly correlated (Pearson correlation $r = 0.9316$ in the FIDELITY dataset). The Pearson correlation between the different risk scores is $r = 0.9879$ in the FIDELITY dataset. The prediction performance is also very similar (concordance index 0.8068 vs. 0.8089 in the model in the paper). Therefore, we conclude that the restriction of markers to the ones available in all dataset is not a major disadvantage.

Table 4: Overview on selected variables for urine data restricted to markers available in all datasets vs. for all biomarkers. The values represent the coefficients in the risk score. ”-” represents that the variable is not part of the respective model. s = serum, u = urine

Trait	Urine model	Full model
Systolic BP	0.18067	0.187534
LDL Ch.	0.01840	0.006535
UACR	0.51748	0.523900
Hematocrit	-	-0.336077
Hemoglobin	-0.33272	-
Albumin (s)	-0.23264	-0.257217
BMI	-0.13045	-0.133432
Creatinine (s)	0.20419	0.201895

3 Supplementary figures

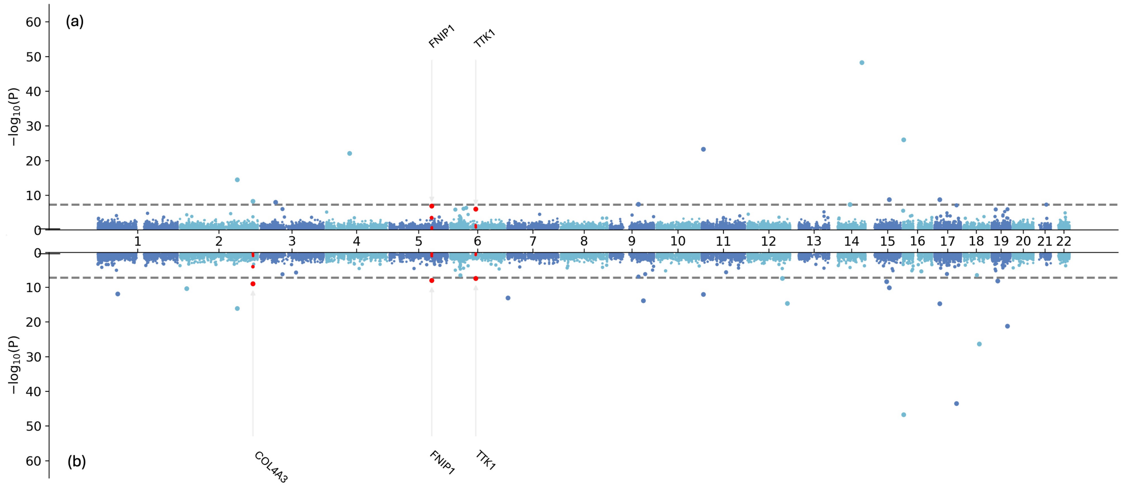

Figure 1: Miami plots for urine (upper panel) and non-urine (lower panel) score for the gene-collapsed analysis. Each dot represents results for one gene.

References

Agarwal, R., Filippatos, G., Pitt, B., Anker, S. D., Rossing, P., Joseph, A., Kolkhof, P., Nowack, C., Gebel, M., Ruilope, L. M., et al. (2022). Cardiovascular and kidney outcomes with finerenone in patients with type 2 diabetes and chronic kidney disease: the fidelity pooled analysis. *European heart journal*, 43(6):474–484.

Bakris, G. L., Agarwal, R., Anker, S. D., Pitt, B., Ruilope, L. M., Rossing, P., Kolkhof, P., Nowack, C., Schloemer, P., Joseph, A., et al. (2020). Effect of finerenone on chronic kidney disease outcomes in type 2 diabetes. *New England journal of medicine*, 383(23):2219–2229.

Bycroft, C., Freeman, C., Petkova, D., Band, G., Elliott, L. T., Sharp, K., Motyer, A., Vukcevic, D., Delaneau, O., O'Connell, J., et al. (2018). The uk biobank resource with deep phenotyping and genomic data. *Nature*, 562(7726):203–209.

Casanova, F., Tyrrell, J., Beaumont, R. N., Ji, Y., Jones, S. E., Hattersley, A. T., Weedon, M. N., Murray, A., Shore, A. C., Frayling, T. M., et al. (2019). A genome-wide association study implicates multiple mechanisms influencing raised urinary albumin–creatinine ratio. *Human Molecular Genetics*, 28(24):4197–4207.

Cheng, J., Novati, G., Pan, J., Bycroft, C., Žemgulytė, A., Applebaum, T., Pritzel, A., Wong, L. H., Zielinski, M., Sargeant, T., Schneider, R. G., Senior, A. W., Jumper, J., Hassabis, D., Kohli, P., and Žiga Avsec (2023). Accurate proteome-wide missense variant effect prediction with alphamissense. *Science*, 381(6664):eadg7492.

Eckardt, K.-U., Bärthlein, B., Baid-Agrawal, S., Beck, A., Busch, M., Eitner, F., Ekici, A. B., Floege, J., Gefeller, O., Haller, H., et al. (2012). The german chronic kidney disease (gckd) study: design and methods. *Nephrology Dialysis Transplantation*, 27(4):1454–1460.

Karczewski, K. J., Francioli, L. C., Tiao, G., Cummings, B. B., Alföldi, J., Wang, Q., Collins, R. L., Laricchia, K. M., Ganna, A., Birnbaum, D. P., Gauthier, L. D., Brand, H., Solomonson, M., Watts, N. A., Rhodes, D., Singer-Berk, M., England, E. M., Seaby, E. G., Kosmicki, J. A., Walters, R. K., Tashman, K., Farjoun, Y., Banks, E., Poterba, T., Wang, A., Seed, C., Whiffin, N., Chong, J. X., Samocha, K. E., Pierce-Hoffman, E., Zappala, Z., O'Donnell-Luria, A. H., Minikel, E. V., Weisburd, B., Lek, M., Ware, J. S., Vittal, C., Armean, I. M., Bergelson, L., Cibulskis, K., Connolly, K. M., Covarrubias, M., Donnelly, S., Ferriera, S., Gabriel, S., Gentry, J., Gupta, N., Jeandet, T., Kaplan, D., Llanwarne, C., Munshi, R., Novod, S., Petrillo, N., Roazen, D., Ruano-Rubio, V., Saltzman, A., Schleicher, M., Soto, J., Tibbetts, K., Tolonen, C., Wade, G., Talkowski, M. E., Aguilar Salinas, C. A., Ahmad, T., Albert, C. M., Ardissino, D., Atzmon, G., Barnard, J., Beaugerie, L., Benjamin, E. J., Boehnke, M., Bonnycastle, L. L., Bottinger, E. P., Bowden, D. W., Bown, M. J., Chambers, J. C., Chan, J. C., Chasman, D., Cho, J., Chung, M. K., Cohen, B., Correa, A., Dabelea, D., Daly, M. J., Darbar, D., Duggirala, R., Dupuis, J., Ellinor, P. T., Elosua, R., Erdmann, J., Esko, T., Färkkilä, M., Florez, J., Franke, A., Getz, G., Glaser, B., Glatt, S. J., Goldstein, D., Gonzalez, C., Groop, L., Haiman, C., Hanis, C., Harms, M., Hiltunen, M., Holi, M. M., Hultman, C. M., Kallela, M., Kaprio, J., Kathiresan, S., Kim, B.-J., Kim, Y. J., Kirov, G., Kooner, J., Koskinen, S., Krumholz, H. M., Kugathasan, S., Kwak, S. H., Laakso, M., Lehtimäki, T., Loos, R. J. F., Lubitz, S. A., Ma, R. C. W., MacArthur, D. G., Marrugat, J., Mattila, K. M., McCarroll, S., McCarthy, M. I., McGovern, D., McPherson, R., Meigs, J. B., Melander, O., Metspalu, A., Neale, B. M., Nilsson, P. M., O'Donovan, M. C., Ongur, D., Orozco, L., Owen, M. J., Palmer, C. N. A., Palotie, A., Park, K. S., Pato, C., Pulver, A. E., Rahman, N., Remes, A. M., Rioux, J. D., Ripatti, S., Roden, D. M., Saleheen, D., Salomaa, V., Samani, N. J., Scharf, J., Schunkert, H., Shoemaker, M. B., Sklar, P., Soininen, H., Sokol, H., Spector, T., Sullivan, P. F., Suvisaari, J., Tai, E. S., Teo, Y. Y., Tiinamaija, T., Tsuang, M., Turner, D., Tusie-Luna, T., Vartiainen, E., Vawter, M. P., Watkins, H., Weersma, R. K., Wessman, M., Wilson, J. G., Xavier, R. J., and Consortium, G. A. D. (2020). The mutational constraint spectrum quantified from variation in 141,456 humans. *Nature*, 581(7809):434–443.

Mbatchou, J., Barnard, L., Backman, J., Marcketta, A., Kosmicki, J. A., Ziyatdinov, A., Benner, C., O'Dushlaine, C., Barber, M., Boutkov, B., Habegger, L., Ferreira, M., Baras, A., Reid, J., Abecasis, G., Maxwell, E., and Marchini, J. (2021). Computationally efficient whole-genome regression for quantitative and binary traits. *Nature Genetics*, 53(7):1097–1103.

McLaren, W., Gil, L., Hunt, S. E., Riat, H. S., Ritchie, G. R. S., Thormann, A., Flicek, P., and Cunningham, F. (2016). The ensembl variant effect predictor. *Genome Biology*, 17(1):122.

Pitt, B., Filippatos, G., Agarwal, R., Anker, S. D., Bakris, G. L., Rossing, P., Joseph, A., Kolkhof, P., Nowack, C., Schloemer, P., et al. (2021). Cardiovascular events with finerenone in kidney disease and type 2 diabetes. *New England Journal of Medicine*, 385(24):2252–2263.

Taliun, D., Harris, D. N., Kessler, M. D., Carlson, J., Szpiech, Z. A., Torres, R., Taliun, S. A. G., Corvelo, A., Gogarten, S. M., Kang, H. M., Pitsillides, A. N., LeFaive, J., Lee, S.-b., Tian, X., Browning, B. L., Das, S., Emde, A.-K., Clarke, W. E., Loesch, D. P., Shetty, A. C., Blackwell, T. W., Smith, A. V., Wong, Q., Liu, X., Conomos, M. P., Bobo, D. M., Aguet, F., Albert, C., Alonso, A., Ardlie, K. G., Arking, D. E., Aslibekyan, S., Auer, P. L., Barnard, J., Barr, R. G., Barwick, L., Becker, L. C., Beer, R. L., Benjamin, E. J., Bielak, L. F., Blangero, J., Boehnke, M., Bowden, D. W., Brody, J. A., Burchard, E. G., Cade, B. E., Casella, J. F., Chalazan, B., Chasman, D. I., Chen, Y.-D. I., Cho, M. H., Choi, S. H., Chung, M. K., Clish, C. B., Correa, A., Curran, J. E., Custer, B., Darbar, D., Daya, M., de Andrade, M., DeMeo, D. L., Dutcher, S. K., Ellinor, P. T., Emery, L. S., Eng, C., Fatkin, D., Fingerlin, T., Forer, L., Fornage, M., Franceschini, N., Fuchsberger, C., Fullerton, S. M., Germer, S., Gladwin, M. T., Gottlieb, D. J., Guo, X., Hall, M. E., He, J., Heard-Costa, N. L., Heckbert, S. R., Irvin, M. R., Johnsen, J. M., Johnson, A. D., Kaplan, R., Kardia, S. L. R., Kelly, T., Kelly, S., Kenny, E. E., Kiel, D. P., Klemmer, R., Konkle, B. A., Kooperberg, C., Köttgen, A., Lange, L. A., Lasky-Su, J., Levy, D., Lin, X., Lin, K.-H., Liu, C., Loos, R. J. F., Garman, L., Gerszten, R., Lubitz, S. A., Lunetta, K. L., Mak, A. C. Y., Manichaikul, A., Manning, A. K., Mathias, R. A., McManus, D. D., McGarvey, S. T., Meigs, J. B., Meyers, D. A., Mikulla, J. L., Minear, M. A., Mitchell, B. D., Mohanty, S., Montasser, M. E., Montgomery, C., Morrison, A. C., Murabito, J. M., Natale, A., Natarajan, P., Nelson, S. C., North, K. E., O'Connell, J. R., Palmer, N. D., Pankratz, N., Peloso, G. M., Peyser, P. A., Pleiness, J., Post, W. S., Psaty, B. M., Rao, D. C., Redline, S., Reiner, A. P., Roden, D., Rotter, J. I., Ruczinski, I., Sarnowski, C., Schoenherr, S., Schwartz, D. A., Seo, J.-S., Seshadri, S., Sheehan, V. A., Sheu, W. H., Shoemaker, M. B., Smith, N. L., Smith, J. A., Sotoodehnia, N., Stilp, A. M., Tang, W., Taylor, K. D., Telen, M., Thornton, T. A., Tracy, R. P., Van Den Berg, D. J., Vasan, R. S., Viaud-Martinez, K. A., Vrieze, S., Weeks, D. E., Weir, B. S., Weiss, S. T., Weng, L.-C., Willer, C. J., Zhang, Y., Zhao, X., Arnett, D. K., Ashley-Koch, A. E., Barnes, K. C., Boerwinkle, E., Gabriel, S., Gibbs, R., Rice, K. M., Rich, S. S., Silverman, E. K., Qasba, P., Gan, W., Abe, N., Almasy, L., Ament, S., Anderson, P., Anugu, P., Applebaum-Bowden, D., Assimes, T., Avramopoulos, D., Barron-Casella, E., Beaty, T., Beck, G., Becker, D., Beitelshees, A., Benos, T., Bezerra, M., Bis, J., Bowler, R., Broeckel, U., Broome, J., Bunting, K., Bustamante, C., Buth, E., Cardwell, J., Carey, V., Carty, C., Casaburi, R., Castaldi, P., Chaffin, M., Chang, C., Chang, Y.-C., Chavan, S., Chen, B.-J., Chen, W.-M., Chuang, L.-M., Chung, R.-H., Comhair, S., Cornell, E., Crandall, C., Crapo, J., Curtis, J., Damcott, C., David, S., Davis, C., Fuentes, L. d. l., DeBaun, M., Deka, R., Devine, S., Duan, Q., Duggirala, R., Durda, J. P., Eaton, C., Ekunwe, L., El Boueiz, A., Erzurum, S., Farber, C., Flickinger, M., Frazar, C., Fu, M., Fulton, L., Gao, S., Gao, Y., Gass, M., Gelb, B., Geng, X. P., Geraci, M., Ghosh, A., Gignoux, C., Glahn, D., Gong, D.-W., Goring, H., Graw, S., Grine, D., Gu, C. C., Guan, Y., Gupta, N., Haessler, J., Hawley, N. L., Heavner, B., Herrington, D., Hersh, C., Hidalgo, B., Hixson, J., Hobbs, B., Hokanson, J., Hong, E., Hoth, K., Hsiung, C. A., Hung, Y.-J., Huston, H., Hwu, C. M., Jackson, R., Jain, D., Jhun, M. A., Johnson, C., Johnston, R., Jones, K., Kathiresan, S., Khan, A., Kim, W., Kinney, G., Kramer, H., Lange, C., Lange, E., Lange, L., Laurie, C., LeBoff, M., Lee, J., Lee, S. S., Lee, W.-J., Levine, D., Lewis, J., Li, X., Li, Y., Lin, H., Lin, H., Lin, K. H., Liu, S., Liu, Y., Liu, Y., Luo, J., Mahaney,

M., and for Precision Medicine (TOPMed) Consortium, N. T.-O. (2021). Sequencing of 53,831 diverse genomes from the nhlbi topmed program. *Nature*, 590(7845):290–299.
